# A Novel Permanent Magnetic Angular Acceleration Sensor

**DOI:** 10.3390/s150716136

**Published:** 2015-07-03

**Authors:** Hao Zhao, Hao Feng

**Affiliations:** 1 College of Information Engineering, Zhejiang University of Technology, Hangzhou 310023, China; E-Mail: zhaohao204@163.com; 2 Nanhu College, Jiaxing University, Jiaxing 314001, China; 3 College of Automation, Hangzhou Dianzi University, Hangzhou 310018, China

**Keywords:** sensor, electromagnetic induction, FEM analysis, calibration and testing

## Abstract

Angular acceleration is an important parameter for status monitoring and fault diagnosis of rotary machinery. Therefore, we developed a novel permanent magnetic angular acceleration sensor, which is without rotation angle limitations and could directly measure the instantaneous angular acceleration of the rotating system. The sensor rotor only needs to be coaxially connected with the rotating system, which enables convenient sensor installation. For the cup structure of the sensor rotor, it has a relatively small rotational inertia. Due to the unique mechanical structure of the sensor, the output signal of the sensor can be directed without a slip ring, which avoids signal weakening effect. In this paper, the operating principle of the sensor is described, and simulated using finite element method. The sensitivity of the sensor is calibrated by torsional pendulum and angle sensor, yielding an experimental result of about 0.88 mV/(rad·s^−2^). Finally, the angular acceleration of the actual rotating system has been tested, using both a single-phase asynchronous motor and a step motor. Experimental result confirms the operating principle of the sensor and indicates that the sensor has good practicability.

## 1. Introduction

Angular acceleration is an important parameter for status monitoring and motion control of rotating system. There exist a number of methods for angular acceleration measuring, including pure mechanical, electromagnetic mechanical, physical, chemical, optical and even radiative methods. Using different measurement methods, different angular acceleration sensors can be developed with different structures and properties. With the development of automatic control technology, the angular acceleration sensor is currently widely used in industry, military, medical science, kinesiology and aerospace, *etc.* [1,2,3,4,5].

Angular acceleration measuring techniques are usually divided into indirect measuring and direct measuring. The indirect acceleration measuring technique are based on analog or digital post processing of available position or velocity signal [6,7,8]; however, the signal processing of these methods are very troublesome, especially the problems of delay characteristics and noise amplification are hard to resolve [9,10,11,12], and also the rotation range is typically limited due to their mechanical structures [13]. All of these have prompted the motivated the efforts to develop transducers for direct sensing of angular acceleration [13,14].

In order to extend the application of angular accelerometers, a series of researches has been carried out on angular acceleration measurement. Mizuno designed and fabricated a bulk silicon micro-machined structure to independently and simultaneously detect angular acceleration and acceleration [15]. An angular accelerometer has been demonstrated on a 6 μm thick silicon on insulator structure with improved cross axis rejection using a dual anchor scheme [16], achieving a sensitivity of 6 mV/r/s^2^ with a resolution of 1 r/s^2^ in a 250 Hz bandwidth. Besides, a six-degree-of-freedom (6-DOF) piezo-resistive accelerometer has been designed by Amarasinghe, which is capable of measuring three components of the angular acceleration on three orthogonal axes at a frequency bandwidth of 300 Hz [17]. Wolfaardt proposed a novel micro-fluidic channel angular accelerometer [18], whose sensor consists of micro-machined spiral channels. It is fabricated on multiple wafers and can be used to construct a spiral-helix fluid column which generates high pressure during angular acceleration around the sensitive axis. In addition, a method for determining the instantaneous angular acceleration of the crankshaft by a magnetic encoder has been presented. This method is based on accurate determination of the measured angular speed and precise values of time when leading edges of individual magnetic teeth pass through the magnetic sensor [19]. Li has proposed a novel micro-electromechanical system (MEMS) pendulum angular accelerometer with electrostatic actuator feedback [20]. Compared with the other MEMS angular accelerometers, the proof pendulum with optimized moment of inertia improves sensitivity and resolution. Moreover, a fiber Bragg grating based angular accelerometer (FBGAA) imposed by an oscillating plate has also been proposed for angular acceleration measurement [21]. A novel wireless thermal convection angular accelerometer without movable parts and grooved cavity has been developed [22], which can be further integrated with an active RFID tag on the same flexible substrate. A novel prototype transducer, which is based on eddy current induction within a moving conductor slab in the presence of permanent magnets, has been developed by Restivo for relative angular acceleration measurement [23]. The contact-less operating principle can be readily applicable to the measurement of linear or angular relative acceleration. A concept of a fiber optic sensor, which consists of a light source, a fiber coil, and a two-beam interferometer, has also been described by Schloeffel for angular acceleration measurement [24].

In this paper, we propose a novel low rotational inertia permanent magnetic angular acceleration sensor. This sensor could directly measure the angular acceleration of the rotating system without rotation angle limitations. The 3D mechanical structure diagram of the sensor is introduced, with its output characterization derived according to the magnetic circuit theorem. The sensor model has been established using finite element method, and the distribution of magnetic flux when the sensor is working has been simulated. By calibration using torsional pendulum and angle sensor, the sensitivity of permanent magnetic sensor is received. Finally, the angular acceleration of the actual rotating systems has been tested, using a single-phase asynchronous motor and step motor, which confirms the operating principle of the sensor.

## 2. Mechanical Structure of the Sensor

**Figure 1:**
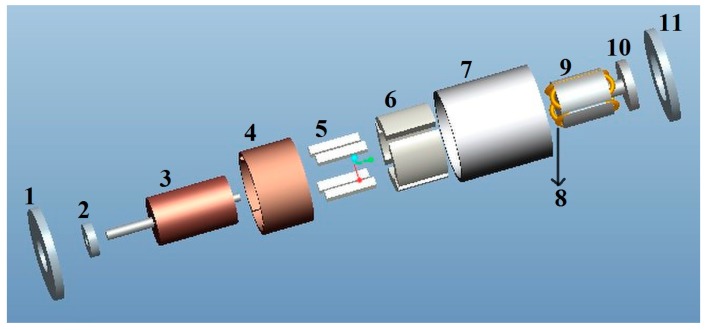
3D assembly diagram of the angular acceleration sensor.

Figure 1 shows a 3D assembly diagram of the permanent magnetic angular acceleration sensor. It components are labeled as front cover 1, bearing 2, cup-shaped rotor 3, copper sleeve 4, permanent magnets 5, outer stator 6, case 7, output winding 8, inner stator 9, base 10, as well as the end cover 11.

The front cover 1, case 7, and end cover 11 are made of ferromagnetic materials. The cup-shaped rotor 3 is a thin-walled non-magnetic cup, which is prepared from high resistivity phosphor bronze, silicon manganese bronze and tin zinc bronze and other materials. The copper sleeve 4 and the base 10 are made of brass with high diamagnetism. The permanent magnets 5 are made using Nd-Fe-B. The outer stator 6 and the inner stator 9 can be made of a high magnetic permeability soft magnetic iron-nickel alloy sheet or high magnetic permeability silicon steel via punching and shearing. The output winding 8 are embedded in the grooves of the inner stator 9.

**Figure 2:**
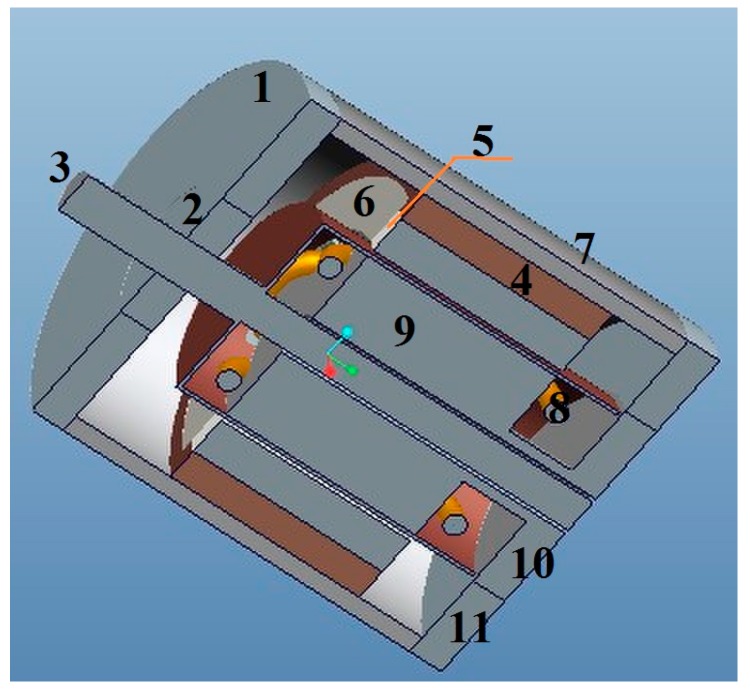
3D cross-sectional view of the angular acceleration sensor.

**Figure 3:**
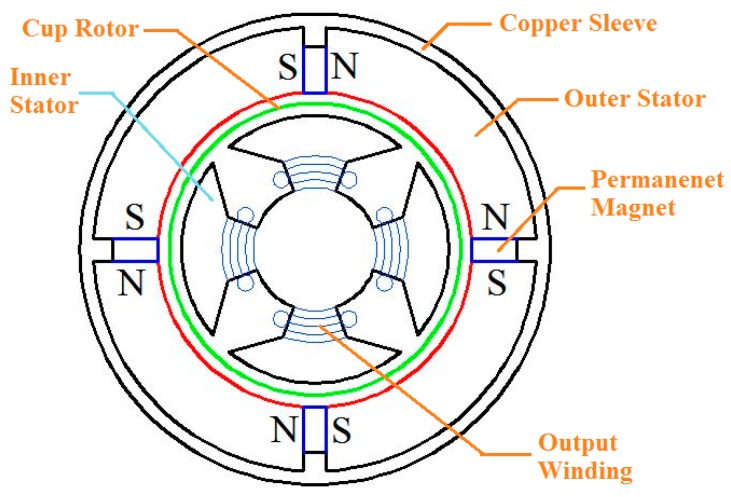
Cross-sectional sketch view of the sensor.

**Figure 4:**
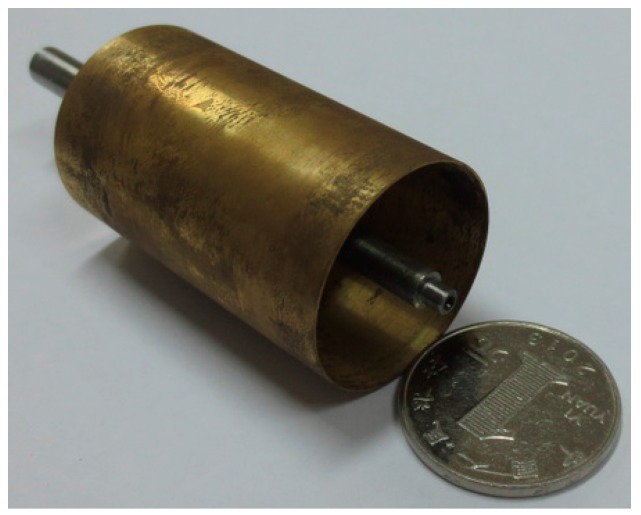
The entity of the cup rotor.

The permanent magnets 5 are used for the generating constant air-gap magnetic field. When measuring the angular acceleration, the cup-shaped rotor 3 is connected coaxially with the shaft of the measured rotating system, and cuts the air-gap magnetic field formed by the permanent magnets 5. The output winding 8 will then generate electrical signals which correspond to the angular acceleration of the rotating system. A 3D cross-sectional view of the sensor is shown in Figure 2, while the cross-sectional sketch map of the sensor is shown in Figure 3. According to the entity of the cup rotor is shown in Figure 4, we could know that the rotational inertia of sensor is relatively small.

## 3. Operating Principle of the Sensor

**Figure 5:**
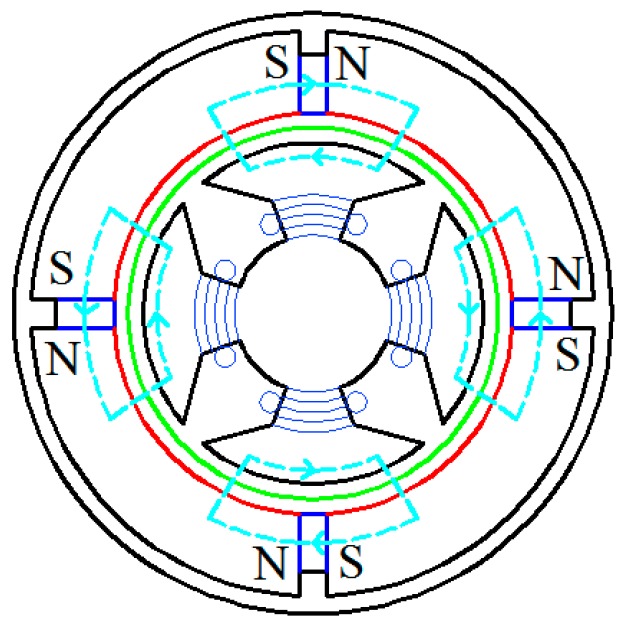
The magnetic circuit of Ф_P_.

The magnetic circuit of Ф_P_ generated by the permanent magnetics is shown in Figure 5. It forms a closed loop by passing through the outer stator, air and the inner stator. The copper sleeve is used here to guarantee Ф_P_ will pass through the air. Assuming symmetrical magnetic circuit, according to the Ohm’s law and Kirchhoff’s voltage law of magnetic circuit theorem, Ф_P_ can be expressed as:

(1) $${\text{Φ}}_{P}=\frac{F_{P}}{R_{mP}}=\frac{H_{P}l_{P}}{R_{mP}}$$

where *F_P_*: The magnetomotive force generated by the permanent magnets; *R_mP_*: The reluctance of magnetic circuit Ф*_P_*; *H_P_*: The strength of the magnetic field in the permanent magnets; *l_P_*: The effective thickness of permanent magnets.

The cup-shaped rotor can be regarded as a squirrel-cage rotor with a large number of bars. When being dragged by the rotating machinery, the cup-shaped rotor will cut Ф*_P_* counterclockwise at a speed of *n* (unit: r/min), generating the electromotive force which is given by:

(2) $$e_{R}=C_{e}{\text{Φ}}_{P}n$$

where *C_e_* is the structure constant of the cup-shaped rotor.

**Figure 6:**
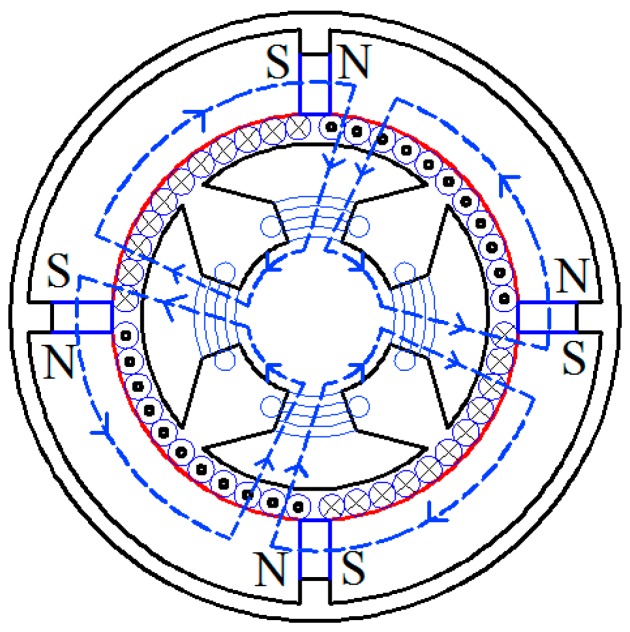
The magnetic circuit of Ф.

The direction of *e_R_* can be determined using the right-hand rule as shown in Figure 6.

Assuming the reactance of cup-shaped rotor is negligible and the resistance is *r_R_*, the current in the cup-shaped rotor *i_R_* is given by:

(3) $$i_{R}=\frac{e_{R}}{r_{R}}$$

According to the right-hand screw rule and the principle that magnetic flux always takes the least resistance path, the magnetic circuit of Ф is shown in Figure 6. It forms a closed loop by passing through the outer stator, the air and the inner stator. According to the Ohm’s law and Kirchhoff’s voltage law of magnetic circuit theorem and assume non-saturated flux, Ф can be expressed as:

(4) $$\text{Φ}=\frac{F}{\sum R_{m}}=\frac{N_{R}i_{R}-H_{P}l_{P}}{\sum R_{m}}$$

where *F*: The magnetomotive force generated by the permanent magnet and the cup rotor; *R_m_*: The reluctance of magnetic circuit Ф; *N_R_*: The effective number of bars of the cup-shaped rotor.

According to the principle of electromagnetic induction, the induction electromotive force in the output winding induced by Ф can be described as

(5) $$e_{o}=-N_{o}\frac{d(2\text{Φ})}{dt}$$

where *N_o_* is the total number of effective coils of the output winding.

Combing Equations (1)–(5), we have

(6) $$e_{o}=\frac{-2N_{o}N_{R}C_{e}{\text{Φ}}_{P}}{r_{R}\cdot \sum R_{m}}\cdot \frac{dn}{dt}$$

As a result, the induced voltage of the output winding is proportional to the measured angular acceleration.

## 4. FEM Modeling and Simulation of the Sensor

FEM is effective in simulating and analyzing the electromagnetic field. Therefore, the sensor model is established using FEM with main structural parameters listed in Table 1.

**Table 1:** Main structural parameters of the sensor.

Component	Material	Inner Diameter	Outer Diameter	Thickness
copper sleeve	Brasses	30 mm	35 mm	5 mm
permanent magnet	XG240/46	22 mm	28 mm	4 mm
Outer stator	DW540_50	22 mm	30 mm	
Cup-shaped rotor	silicon manganese bronze	21 mm	21.5 mm	0.5 mm
Inner stator	DW540_50	10 mm	20.5 mm	
Output winding	Copper-75C		5 mm	

**Figure 7:**
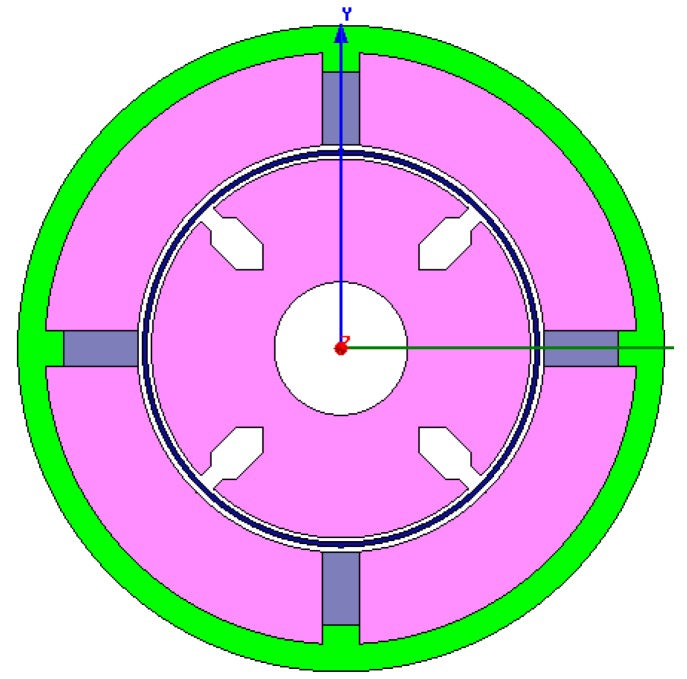
The sensor model established using finite element method.

**Figure 8:**
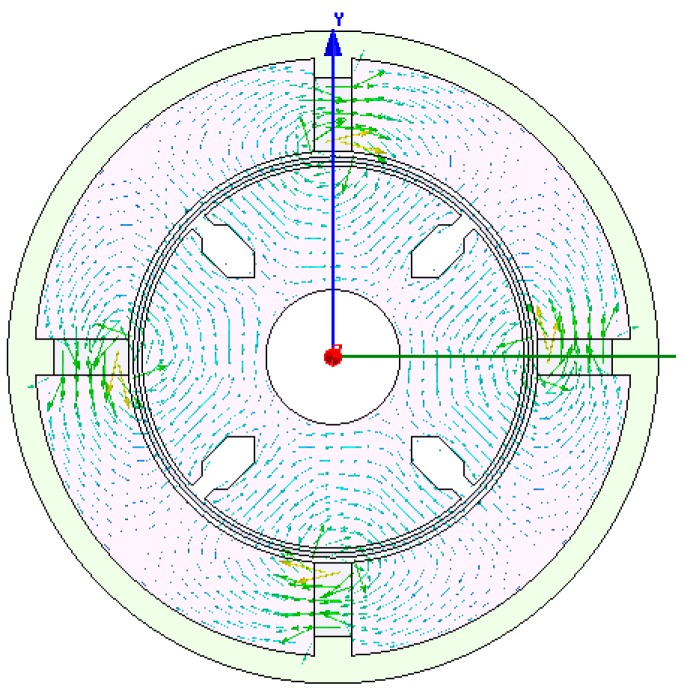
Distribution of magnetic flux Ф_P_.

**Figure 9:**
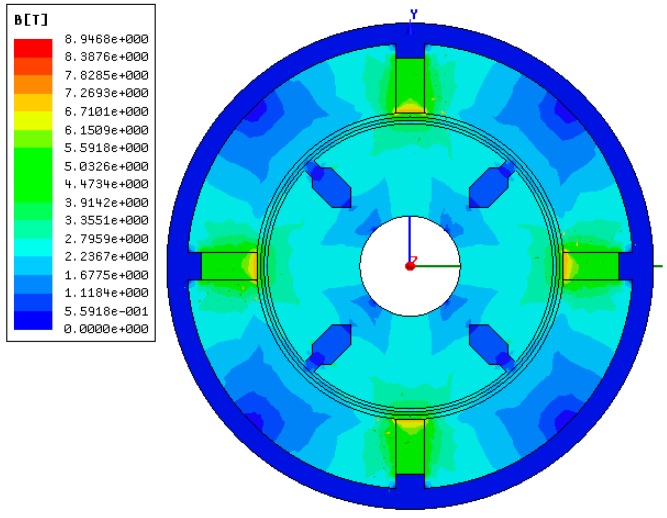
Density cloud distribution of flux Ф_P_.

The two dimensional structure of the sensor is modeled as shown Figure 7. The remanence of permanent magnet is 1.25 T, the coercive force of permanent magnet is −94,700 A/m, and the mesh length is set to 0.5 mm. After setting the boundary conditions, simulations are performed to obtain the distribution of Ф_P_ (shown in Figure 8) and the density cloud distribution of Ф_P_ (shown in Figure 9).

According to Figure 8, the simulated Ф_P_ distribution is consistent with that in Figure 5, and there is no flux crossed with the output winding. According to Figure 9, the magnetic circuits are not saturated.

**Figure 10:**
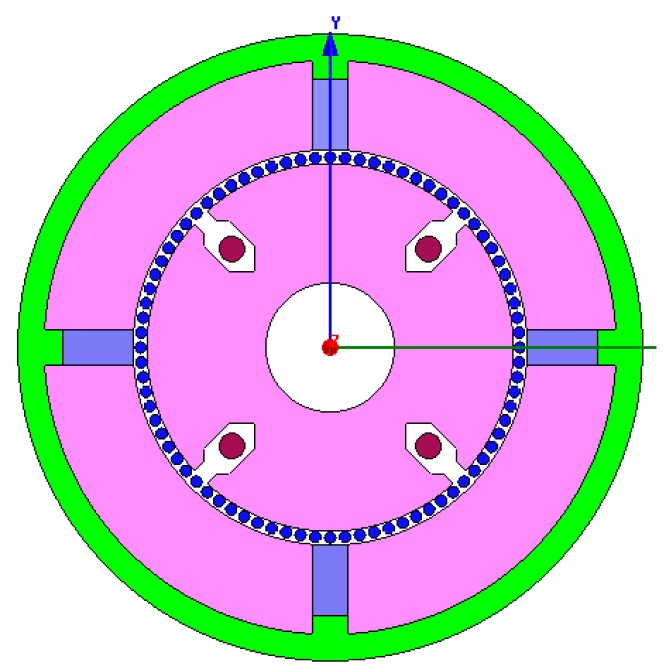
The cup rotor regarded as a squirrel-cage rotor.

**Figure 11:**
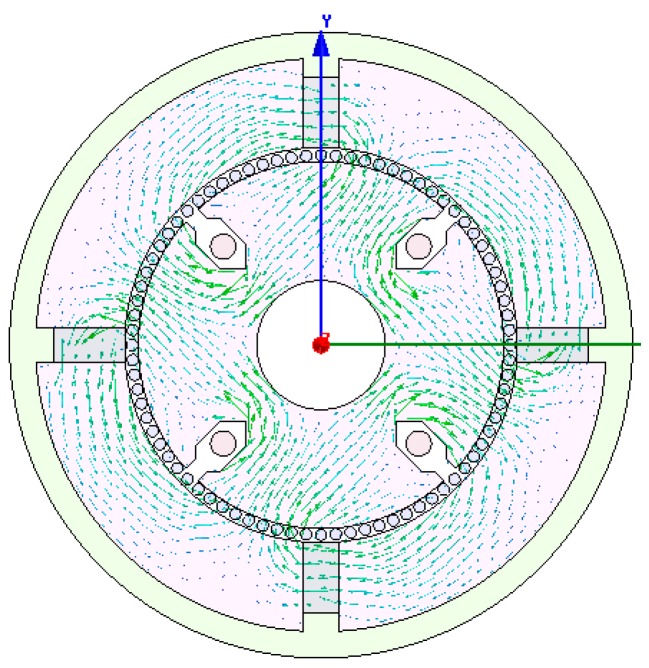
Distribution of magnetic flux Ф.

**Figure 12:**
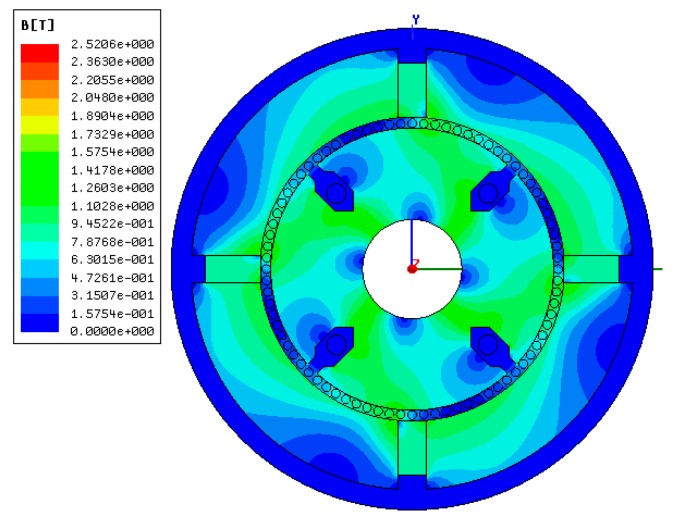
Density cloud distribution of flux Ф.

The cup-shaped rotor can be regarded as a squirrel-cage rotor with a large number of bars as shown in Figure 10. The distribution of Ф is shown in Figure 11, which is consistent with the result shown in Figure 6, and the density cloud distribution of Ф is shown in Figure 12.

**Figure 13:**
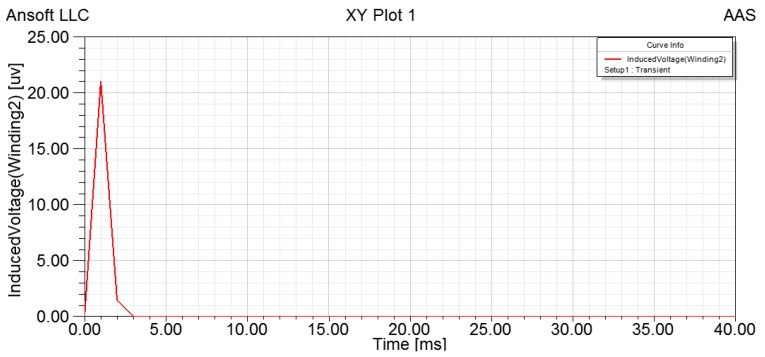
Induced voltage of output winding without angular acceleration.

Assuming constant angular velocity, *i.e.*, no angular acceleration and *n* = *n*_m_, we have constant *i_R_* generated by the electromotive force *e_R_*. Let *i_R_* = 0.5 A, the simulation results obtained are shown in Figure 13.

As we can see in Figure 13, there is a pulse which peaks at 21 μV between 0.00 and 3.00 ms. This small peak is due to the instant increase of *n* from 0 to *n_m_*. With a constant angular velocity, the induced voltage of angular acceleration sensor is zero, which confirms the operating principle of the angular acceleration sensor.

Assuming the angular velocity change follows a sine wave and its direction stays constant, we have

(7) $$n=n_{m}+n_{m}\sin(2\pi \cdot 50\cdot t)$$

As a result, *i_R_* generated by the electromotive force is given by:

(8) $$i_{R}=i_{m}+i_{m}\sin(2\pi \cdot 50\cdot t)$$

**Figure 14:**
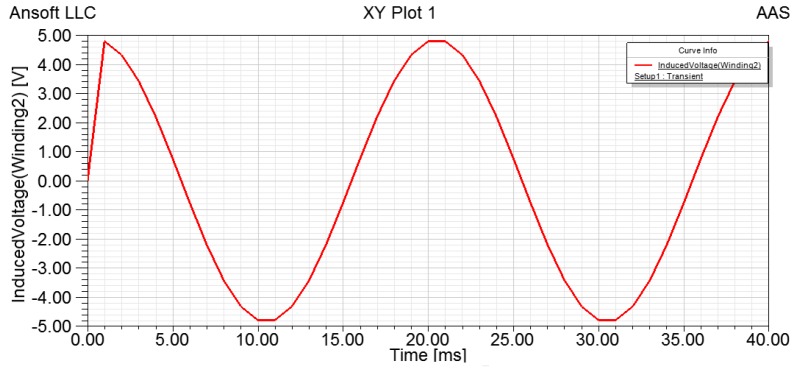
Induced voltage of output winding with angular acceleration.

Let *i_m_* = 0.5 A, the simulation results obtained are shown in Figure 14.

Similarly, we could observe a pulse which peaks at 4.9 V at 1.00 ms due to the speed change and the angular acceleration is given by:

(9) $$\frac{dn}{dt}=100\cdot \pi \cdot n_{m}\cos(2\pi \cdot 50\cdot t)$$

As a result, the change of the induced voltage of the sensor follows a cosine wave, which verifies the operating principle of the sensor.

## 5. Calibration and Angular Acceleration Testing Experiments

### 5.1. The Composition and Principle of the Calibration Equipment

**Figure 15:**
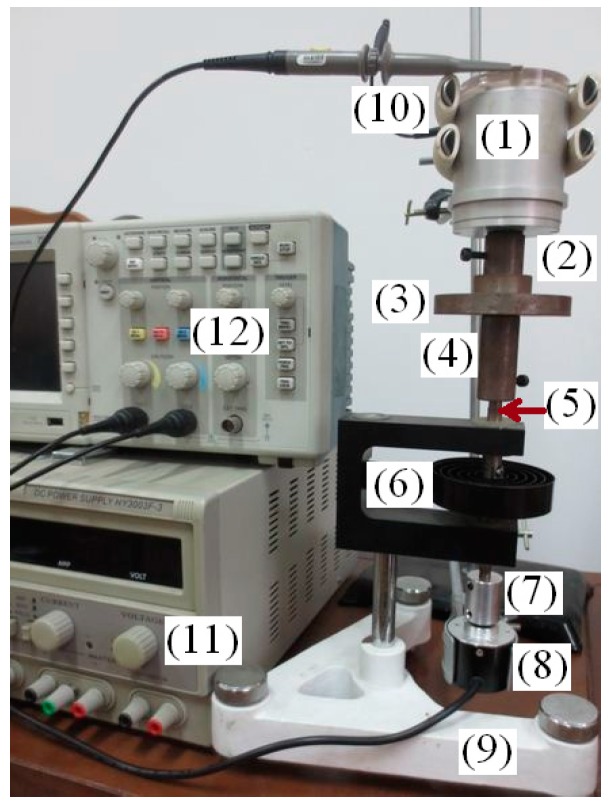
The calibration devices of angular acceleration sensor.

The sensitivity of the angular acceleration sensor is calibrated by torsional pendulum and angle sensor, as shown in Figure 15. The calibration devices include: (1) angular acceleration sensor, (2) coupling, (3) mass, (4) coupling, (5) torsion bar, (6) coil spring, (7) coupling, (8) angle sensor, (9) base, (10) bracket, (11) DC power and (12) digital oscilloscope.

According to the physics laws of pendulum, when an initial force is applied to the torsion bar, the coil spring will rotate angle θ to produce the restoring torque, which can be described as

(10) $$T_{1}=K\theta$$

where *K* is the stiffness factor of the coil spring.

The system will follow a simple harmonic motion when the initial force disappears, in which the system is also affected by the air resistance torque. Therefore, we have:

(11) $$T_{2}=\alpha \frac{d\theta }{dt}$$

where α is the drag torque coefficient.

According to torque balance equation, we have

(12) $$T_{1}+T_{2}=J\frac{d^{2}\theta }{dt^{2}}$$

where *J* is the rotational inertia of calibration system.

Combining Equations (10)–(12), we have

(13) $$J\frac{d^{2}\theta }{dt^{2}}+\alpha \frac{d\theta }{dt}+K\theta =0$$

Let $2\beta =\frac{\alpha }{J}$, $\omega_{n}^{2}=\frac{k}{J}$, we have

(14) $$\frac{d^{2}\theta }{dt^{2}}+2\beta \frac{d\theta }{dt}+\omega_{n}^{2}\theta =0$$

where β and *ω_n_* are the damping ratio coefficient and the intrinsic oscillation angular frequency of the calibration system, respectively.

Solving Equation (14), we have

(15) $$\theta (t)=\frac{\theta_{0}}{\sqrt{1-\xi^{2}}}e^{-\beta t}\cos(\omega_{d}t-\phi )$$

where θ_0_ is the initial amplitude of the pivot angle, $\omega_{d}=\omega_{n}\sqrt{1-\xi^{2}}$ is the damped oscillation angular frequency and the damping ratio is $\xi \omega_{n}=\beta$.

According to the rule of simple harmonic motion, the period of motion *t* is given by

(16) $$t=\frac{2\pi }{\omega_{d}}=\frac{2\pi }{\omega_{n}\sqrt{1-\xi^{2}}}=\frac{2\pi }{\sqrt{\omega_{n}^{2}-\beta^{2}}}$$

Combining Equation (16) and $\omega_{n}^{2}=\frac{k}{J}$, we have

(17) $$\frac{K}{J}=\frac{4\pi^{2}}{t^{2}}+\beta^{2}$$

From Equation (15), we can derive

(18) $$\frac{\theta_{0}}{\theta_{N}}=e^{\beta Nt}$$

where θ_0_ is the amplitude of the initial angle and θ*_N_* is the amplitude of the *N*th cycle, yielding

(19) $$\beta =\frac{1}{Nt}\ln\frac{\theta_{0}}{\theta_{N}}$$

The angular velocity of the pendulum is $\frac{d\theta }{dt}=0$ when the coil spring is at the amplitude position, which simplifies Equation (14) into

(20) $$J\frac{d^{2}\theta }{dt^{2}}+K\theta =0$$

Combining Equations (17), (19) and (20), we have

(21) $$\frac{d^{2}\theta }{dt^{2}}=-\frac{k}{J}\theta =-(\frac{4\pi^{2}}{T^{2}}+\beta^{2})\theta =-[\frac{4\pi^{2}}{T^{2}}+{\left(\frac{1}{Nt}\ln\frac{\theta_{0}}{\theta_{N}}\right)}^{2}]\theta$$

where the cycle *t* and the rotor angle θ can be obtained from the output waveform of angle sensor detected by the oscilloscope. From Equation (21), the actual angular acceleration of the system can be obtained, which is corresponding to the output voltage of the angular acceleration sensor. Additionally, we can obtain the sensitivity coefficient of the angular acceleration sensor.

### 5.2. The Calibration Experiment Results

**Figure 16:**
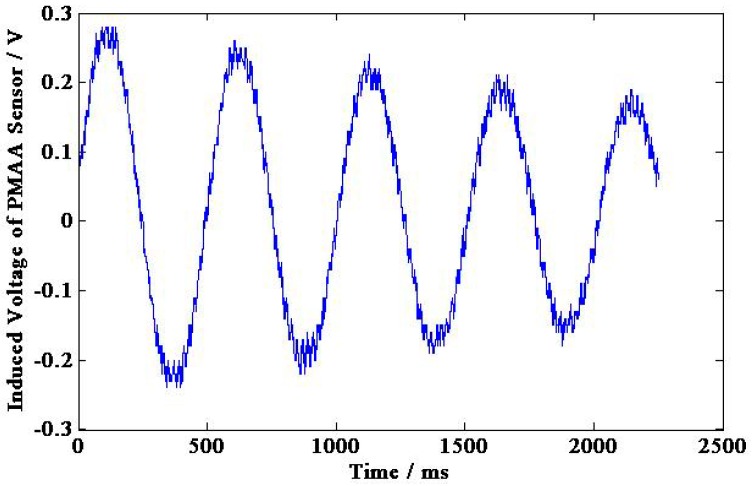
Induced voltage of the permanent magnetic angular acceleration sensor.

**Figure 17:**
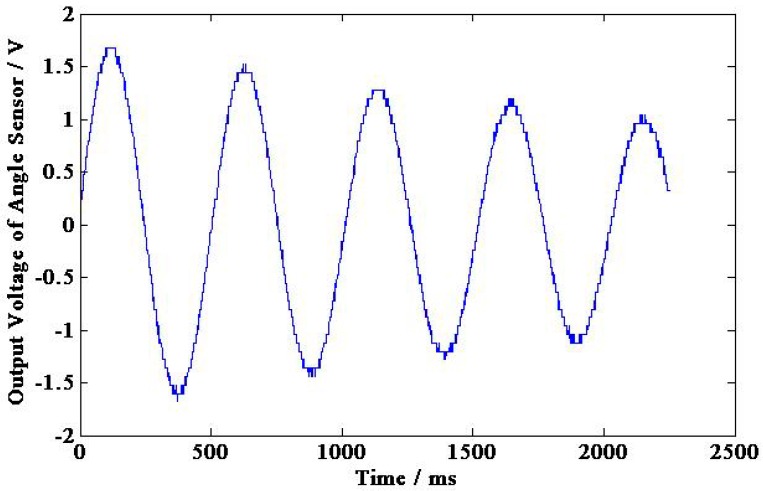
Output voltage of the angle sensor.

The output voltage of permanent magnetic angular acceleration sensor and angle sensor are shown in Figure 16 and Figure 17, respectively, and the experimental calibration data are shown in Table 2. According to Table 2 and Equation (21), the sensitivity of the permanent magnetic angular acceleration sensor is about 0.88 mV/(rad·s^−2^).

**Table 2:** Experimental calibration data.

The peak voltage of the PMAA sensor (V)	0.28	0.25	0.22	0.20	0.17
The peak voltage of angle sensor (V)	1.68	1.44	1.28	1.12	1.04
The time corresponding peak (s)	0.11	0.60	1.13	1.65	2.14
Oscillation period *t*/(s)	(0.49 + 0.53 + 0.52 + 0.49)/4 = 0.5075				
Angle values (rad)	2.11	1.809	1.608	1.407	1.307
System damping ratio coefficient β	(ln(2.11 ÷ 1.307)) ÷ 4 ÷ 0.5075 = 0.2359				
Angular acceleration value (rad/s^2^)	322.4	276.4	245.7	215	199.7
Sensitivity coefficient (mV/(rad/s^2)^)	0.87	0.90	0.89	0.93	0.85

### 5.3. Single-Phase Asynchronous Motor Angular Acceleration Testing

**Figure 18:**
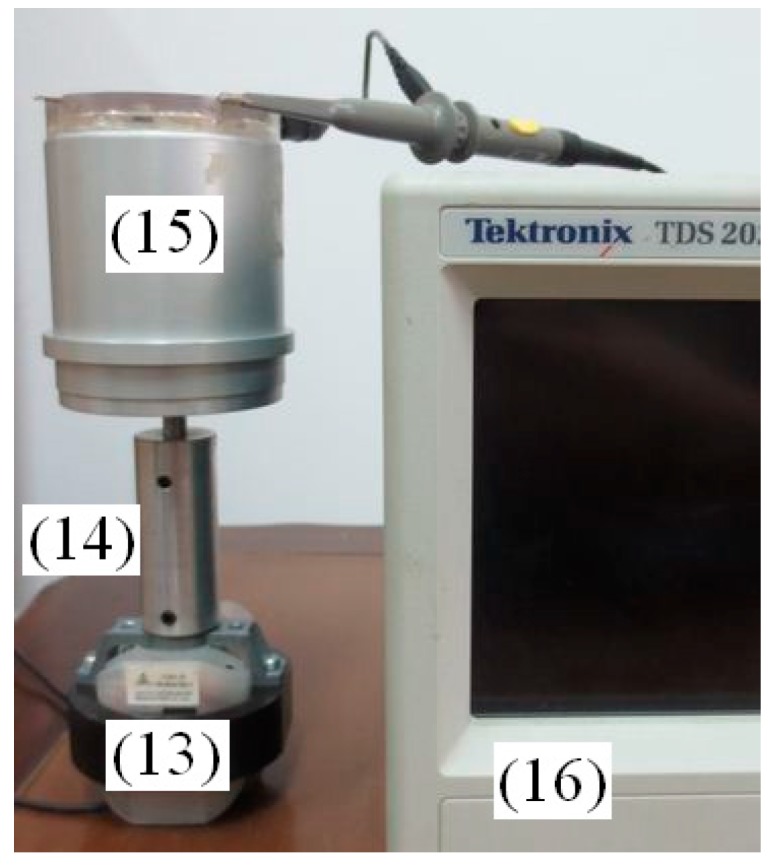
Connection diagram of a single-phase asynchronous motor angular acceleration testing.

**Figure 19:**
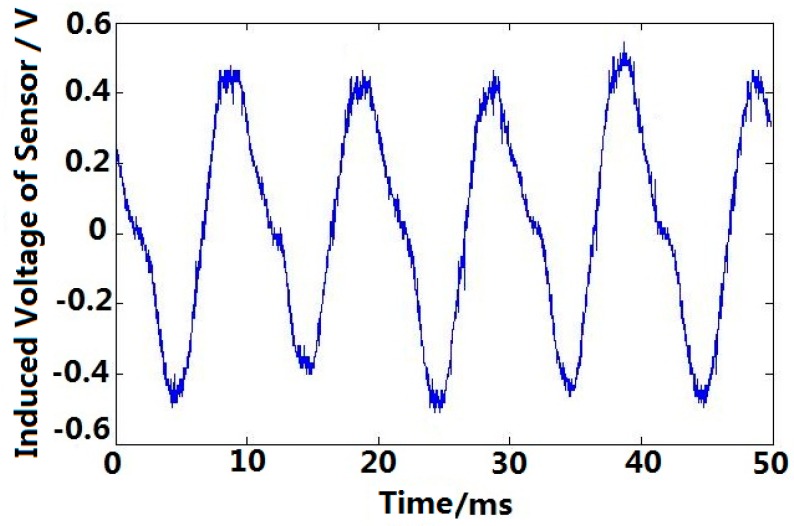
Angular acceleration of a single-phase asynchronous motor.

The rotating machine is a single-phase asynchronous motor with *P*_N_ = 120 W, *U*_N_ = 220 V, *I*_N_ = 1 A, and *n*_N_ = 1450 r/min. The cup-shaped rotor of sensor is connected with the rotating system coaxially. As shown in Figure 18, different components are labeled as (13) single-phase asynchronous motor, (14) coupling, (15) angular acceleration sensor and (16) digital oscilloscope. The induced voltage of angular acceleration sensor output winding are shown in Figure 19.

There exists obvious angular acceleration when the single-phase asynchronous motor is working, which can be detected by the sensor, and the frequency of angular acceleration is about 100 Hz, which is doubled as excitation voltage frequency.

The angular acceleration generated by single-phase asynchronous motor is mainly due to the existence of an ellipse rotating magnetic field generated by the motor stator. As the amplitude and the velocity of the ellipse rotating magnetic field changes continuously, the current induced in the rotor bar of the single-phase asynchronous motor will also keep changing. The current, coupled with the stator ellipse rotating magnetic field, will then generate the electromagnetic driver torque of motor, which eventually lead to the angular acceleration when the rotor is in operation. 

And according to the decomposition theory of rotating magnetic field, the elliptical rotating magnetic field can be decomposed into two circular rotating magnetic fields, their amplitudes are not equal and directions are opposite. The forward circular rotating magnetic field couple with the rotor current induced by the reverse circular rotating magnetic field, and the reverse circular rotating magnetic field couple with the rotor current induced by the forward circular rotating magnetic field, the pulsating torque which frequency is doubled as excitation voltage frequency is produced, and the corresponding experiment results are shown as Figure 19.

### 5.4. Step Motor Angular Acceleration Testing

**Figure 20:**
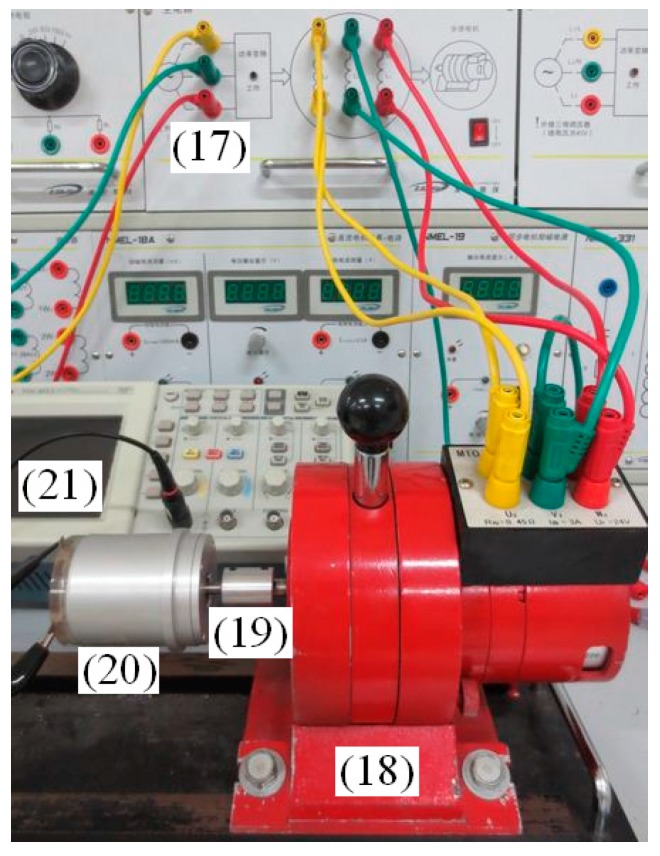
Connection diagram of step motor angular acceleration testing. (*f* = 702 Hz).

**Figure 21:**
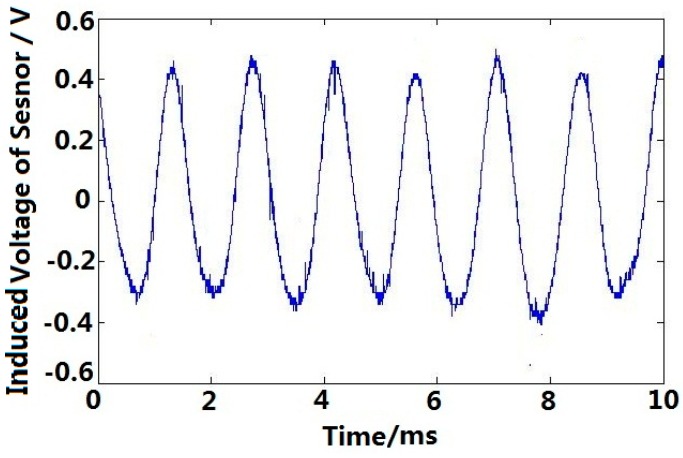
Angular acceleration in continuous operation mode.

The rotating machine is a three-phase step motor, whose resistance of each winding is 45 Ω. The cup-shaped rotor of sensor is connected with the motor coaxially. As shown in Figure 20, each component is labeled as (17) step motor drive controller, (18) step motor, (19) coupling, (20) angular acceleration sensor and (21) digital oscilloscope. Figure 21 shows the induced voltage of the angular acceleration sensor when the step motor in continuous operation mode (pulse frequency *f* = 702 Hz).

Obvious angular acceleration can be observed when the step motor is in continuous operation mode, which confirms the operating principle of the angular acceleration sensor. For the step motor, the drive torque change follows a sine wave according to the torque-angle characteristic, and the rotating magnetic field generated by stator is in jumping mode, both of which cause the angular acceleration in the step motor. And according to the operation principle of step motor, the rotor turning one step when one pulse going through stator winding, then one cycle corresponding angular acceleration signal is produced, so the frequency of angular acceleration sensor output signal, which is same as the frequency of excitation pulses in stator winding, as shown in Figure 21.

### 5.5. Practicability Improvements of Angular Acceleration Sensor

If a magnetic field is generated, either by the rotating machine, for example, the step motor or the single-phase asynchronous motor in this experiments, it will couple with the output winding of the angular acceleration sensor, eventually causing noise in the induced voltage of the angular acceleration sensor.

**Figure 22:**
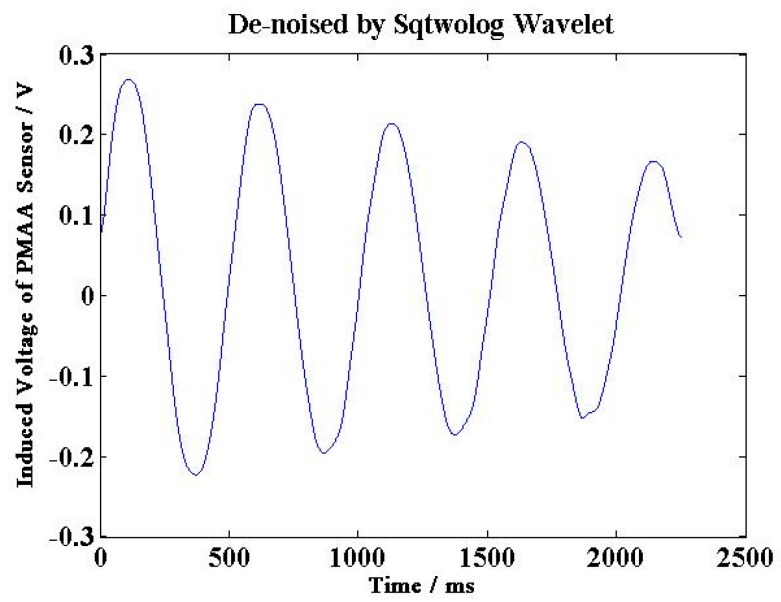
The sensor output signals de-noised by sqtwolog wavelet.

In order to improve the practical performance of sensor, the noise in the output signal can be reduced by fabricating the case and covers of the sensor using materials with high magnetic shielding performance, or developing a filtering algorithm (e.g., Wavelet de-noising algorithm) as shown in Figure 22, the output signal of this angular acceleration sensor is de-noised by Sqtwolog Wavelet, and the noise is significantly reduced compared with Figure 16.

## 6. Conclusions

In this paper, we propose a novel and practical permanent magnetic angular acceleration sensor, which could directly measure the instantaneous angular acceleration of the rotating system, so there is no more problems of delay characteristics and noise amplification compared with indirect angular acceleration measuring techniques [9,10,11,12,19]. More importantly, the sensor structure is simple and the sensor rotor only needs to be coaxially connected to the rotating system, which enables convenient sensor installation compared with micro-mechanical silicon angular acceleration sensor [15,16,20]. In addition, the sensor without rotation angle limitations, and the output signal of the sensor can be directed without a slip ring, which avoids signal weakening effect and shows high disturbance resistance compare with piezo-resistive accelerometer [17] and micro-fluidic angular accelerometer [18].

To extend the application of angular acceleration sensors, future work will aim at applying the developed sensor in the status monitoring and fault diagnosis of rotating machinery and complicated transmission equipment.
